# (*E*)-1-(4-Chloro­benzyl­idene)thio­semi­carbazide

**DOI:** 10.1107/S1600536811004120

**Published:** 2011-02-19

**Authors:** Aamer Saeed, Shams-ul Mahmood, Michael Bolte, Abdul Rauf, Mohammad Subhan

**Affiliations:** a Department of Chemistry, Quaid-i-Azam University, Islamabad 45320, Pakistan; b Institut für Anorganische Chemie, J. W. Goethe-Universität Frankfurt, Max-von-Laue-Strasse 7, 60438 Frankfurt/Main, Germany; c Department of Chemistry, Islamia Islamia College University, Peshawar, Pakistan; d Department of Chemistry, Islamia University, Bahawalpur, Pakistan

## Abstract

In the crystal of the title compound, C_8_H_8_ClN_3_S, mol­ecules are connected by N—H⋯S hydrogen bonds into strips parallel to the (112) planes and running along [1
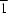
0]. One of the amino H atoms is not involved in a classical hydrogen bond. In addition, there is a rather short inter­molecular Cl⋯S distance of 3.3814 (5) Å.

## Related literature

For background to Schiff bases, see: Mobinikhaledi *et al.* (2010 ▶); Hamaker *et al.* (2010 ▶); Mirkhani *et al.* (2010 ▶); Thangadurai *et al.* (2002 ▶). Ji & Lu (2010*a* ▶,*b* ▶); Lü *et al.* (2008 ▶). For a related structure, see: Zhang & Li (2008 ▶). For bioactivity, see: Chohan *et al.* (2004 ▶).
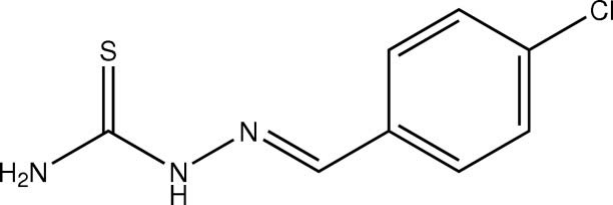


## Experimental

### 

#### Crystal data

C_8_H_8_ClN_3_S
*M* *_r_* = 213.68
Triclinic, 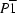

*a* = 5.7611 (5) Å
*b* = 7.8329 (7) Å
*c* = 11.2016 (10) Å
α = 83.852 (7)°
β = 75.373 (7)°
γ = 76.353 (7)°
*V* = 474.69 (7) Å^3^
*Z* = 2
Mo *K*α radiation
μ = 0.58 mm^−1^
*T* = 173 K
0.38 × 0.35 × 0.27 mm

#### Data collection

Stoe IPDS II two-circle diffractometer
Absorption correction: multi-scan (*MULABS*; Spek, 2009; ▶ Blessing, 1995 ▶) *T*_min_ = 0.811, *T*_max_ = 0.860
8657 measured reflections
2179 independent reflections
2054 reflections with *I* > 2σ(*I*)
*R*_int_ = 0.055

#### Refinement

*R*[*F* ^2^ > 2σ(*F* ^2^)] = 0.029
*wR*(*F* ^2^) = 0.079
*S* = 1.10
2179 reflections
131 parameters
H atoms treated by a mixture of independent and constrained refinement
Δρ_max_ = 0.39 e Å^−3^
Δρ_min_ = −0.27 e Å^−3^

### 

Data collection: *X-AREA* (Stoe & Cie, 2001 ▶); cell refinement: *X-AREA*; data reduction: *X-AREA*; program(s) used to solve structure: *SHELXS97* (Sheldrick, 2008 ▶); program(s) used to refine structure: *SHELXL97* (Sheldrick, 2008 ▶); molecular graphics: *XP* (Sheldrick, 2008 ▶); software used to prepare material for publication: *SHELXL97*.

## Supplementary Material

Crystal structure: contains datablocks global, I. DOI: 10.1107/S1600536811004120/bg2386sup1.cif

Structure factors: contains datablocks I. DOI: 10.1107/S1600536811004120/bg2386Isup2.hkl

Additional supplementary materials:  crystallographic information; 3D view; checkCIF report

## Figures and Tables

**Table 1:** Hydrogen-bond geometry (Å, °)

*D*—H⋯*A*	*D*—H	H⋯*A*	*D*⋯*A*	*D*—H⋯*A*
N2—H2⋯S1^i^	0.863 (19)	2.628 (19)	3.4288 (12)	154.7 (16)
N3—H3*A*⋯S1^ii^	0.877 (19)	2.65 (2)	3.5119 (12)	170.1 (17)

Symmetry codes: (i) 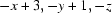; (ii) 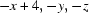.

**Fig. 1.**
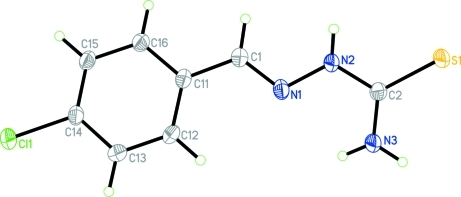
Molecular structure of title compound. Displacement ellipsoids are drawn at the 50% probability level.

**Fig. 2.**
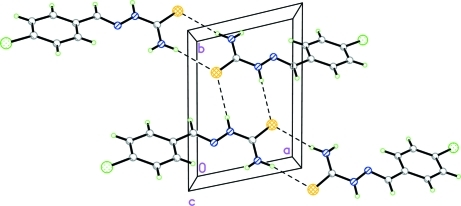
Crystal packing with intermolecular hydrogen bonds indicated as dashed lines.

## supplementary crystallographic information

### Comment

Schiff bases derived from semicarbazide and aromatic aldehydes are an important class of organic compounds because of their applications in many fields including inorganic, biological, and analytical chemistry (Mobinikhaledi *et al.*, 2010; Thangadurai *et al.*, 2002). Schiff bases are known to be versatile ligands in coordination chemistry (Ji & Lu, 2010*a*,2010*b*; Hamaker *et al.*, 2010; Mirkhani *et al.*, 2010). Shiff bases and their complexes are also important in material science applications like organic light emitting diodes (Lü *et al.*, 2008).

The title compound features an essentially planar molecule: the largest deviation of a torsion angle from 0 or 180Å is 13.02 (19)° for N1—C1—C11—C12. Bond lengths and angles do not show any unusual values. In the crystal, the molecules are connected by N—H···S hydrogen bonds into strips parallel to the (1 1 2) planes and running along [1 1 0]. One of the amino H atoms is not involved in a classical hydrogen bond. In addition there is a rather short intermolecular Cl···S^i^ distance of 3.3814 (5)Å (symmetry operator (i): *x* - 2, *y*, *z* + 1).

### Experimental

Semicarbazide, 0.137 g (1 mmol) dissolved in 5 ml e thanol was added dropwise to a solution of 4-chlorobenzaldehyde, 0.122 g (1.1 mmol) in 10 ml e thanol at room temperature with continuous stirring. The reaction mixture was refluxed for 4 h and completion monitored by TLC. The reaction mixture was concentrated and the resulting product was separated. Colourless single crystals of the compound, suitable for X-ray crystallography, were grown by slow evaporation from a (2:1) ethyl acetate-ethanol solution. Anal.: calcd. for C_8_H_8_N_3_SCl: C, 44.95; H, 3.74; N, 19.66; S, 14.98%; found: C, 45.01; H, 3.61; N, 19.81; S, 14.83%.

### Refinement

H atoms bonded to N were freely refined. H atoms bonded to C were geometrically positioned and refined using a riding model with C—H = 0.95Å and U(H) set to 1.2*U*_eq_(C).

### Crystal data

**Table:**

C_8_H_8_ClN_3_S	*Z* = 2
*M**_r_* = 213.68	*F*(000) = 220
Triclinic, *P*1	*D*_x_ = 1.495 Mg m^−^^3^
Hall symbol: -P 1	Mo *K*α radiation, λ = 0.71073 Å
*a* = 5.7611 (5) Å	Cell parameters from 7492 reflections
*b* = 7.8329 (7) Å	θ = 3.4–27.8°
*c* = 11.2016 (10) Å	µ = 0.58 mm^−^^1^
α = 83.852 (7)°	*T* = 173 K
β = 75.373 (7)°	Block, colourless
γ = 76.353 (7)°	0.38 × 0.35 × 0.27 mm
*V* = 474.69 (7) Å^3^	

### Data collection

**Table:**

Stoe IPDS II two-circle diffractometer	2179 independent reflections
Radiation source: fine-focus sealed tube	2054 reflections with *I* > 2σ(*I*)
graphite	*R*_int_ = 0.055
ω scans	θ_max_ = 27.7°, θ_min_ = 3.4°
Absorption correction: multi-scan (*MULABS*; Spek, 2009; Blessing, 1995)	*h* = −7→7
*T*_min_ = 0.811, *T*_max_ = 0.860	*k* = −10→10
8657 measured reflections	*l* = −14→14

### Refinement

**Table:**

Refinement on *F*^2^	Secondary atom site location: difference Fourier map
Least-squares matrix: full	Hydrogen site location: inferred from neighbouring sites
*R*[*F*^2^ > 2σ(*F*^2^)] = 0.029	H atoms treated by a mixture of independent and constrained refinement
*wR*(*F*^2^) = 0.079	*w* = 1/[σ^2^(*F*_o_^2^) + (0.0425*P*)^2^ + 0.1107*P*] where *P* = (*F*_o_^2^ + 2*F*_c_^2^)/3
*S* = 1.10	(Δ/σ)_max_ = 0.001
2179 reflections	Δρ_max_ = 0.39 e Å^−^^3^
131 parameters	Δρ_min_ = −0.27 e Å^−^^3^
0 restraints	Extinction correction: *SHELXL97* (Sheldrick, 2008), Fc^*^=kFc[1+0.001xFc^2^λ^3^/sin(2θ)]^-1/4^
Primary atom site location: structure-invariant direct methods	Extinction coefficient: 0.061 (7)

### Special details

**Table:**

Geometry. All e.s.d.'s (except the e.s.d. in the dihedral angle between two l.s. planes) are estimated using the full covariance matrix. The cell e.s.d.'s are taken into account individually in the estimation of e.s.d.'s in distances, angles and torsion angles; correlations between e.s.d.'s in cell parameters are only used when they are defined by crystal symmetry. An approximate (isotropic) treatment of cell e.s.d.'s is used for estimating e.s.d.'s involving l.s. planes.
Refinement. Refinement of *F*^2^ against ALL reflections. The weighted *R*-factor *wR* and goodness of fit *S* are based on *F*^2^, conventional *R*-factors *R* are based on *F*, with *F* set to zero for negative *F*^2^. The threshold expression of *F*^2^ > σ(*F*^2^) is used only for calculating *R*-factors(gt) *etc*. and is not relevant to the choice of reflections for refinement. *R*-factors based on *F*^2^ are statistically about twice as large as those based on *F*, and *R*- factors based on ALL data will be even larger.

### Fractional atomic coordinates and isotropic or equivalent isotropic displacement parameters (Å^2^)

**Table:**

	*x*	*y*	*z*	*U*_iso_*/*U*_eq_	
Cl1	0.16691 (5)	0.24404 (4)	0.64870 (3)	0.02702 (12)	
S1	1.79101 (5)	0.26359 (4)	−0.06509 (3)	0.02625 (12)	
N1	1.18834 (19)	0.23473 (14)	0.18778 (10)	0.0215 (2)	
N2	1.35359 (19)	0.28547 (14)	0.08531 (10)	0.0220 (2)	
H2	1.322 (3)	0.392 (3)	0.0550 (17)	0.033 (4)*	
N3	1.6366 (2)	0.03361 (14)	0.11273 (11)	0.0256 (2)	
H3A	1.787 (3)	−0.030 (3)	0.0942 (17)	0.037 (5)*	
H3B	1.529 (4)	0.002 (2)	0.1674 (19)	0.037 (5)*	
C1	0.9739 (2)	0.33723 (16)	0.20735 (11)	0.0206 (2)	
H1	0.9408	0.4333	0.1503	0.025*	
C2	1.5844 (2)	0.18669 (15)	0.05230 (11)	0.0200 (2)	
C11	0.7791 (2)	0.30948 (15)	0.31550 (11)	0.0194 (2)	
C12	0.8261 (2)	0.19449 (17)	0.41513 (12)	0.0258 (3)	
H12	0.9887	0.1297	0.4129	0.031*	
C13	0.6388 (2)	0.17389 (17)	0.51702 (12)	0.0275 (3)	
H13	0.6721	0.0962	0.5847	0.033*	
C14	0.4012 (2)	0.26838 (16)	0.51905 (11)	0.0209 (2)	
C15	0.3492 (2)	0.38356 (16)	0.42174 (11)	0.0221 (2)	
H15	0.1859	0.4470	0.4241	0.027*	
C16	0.5394 (2)	0.40491 (16)	0.32075 (11)	0.0216 (2)	
H16	0.5060	0.4855	0.2544	0.026*	

### Atomic displacement parameters (Å^2^)

**Table:**

	*U*^11^	*U*^22^	*U*^33^	*U*^12^	*U*^13^	*U*^23^
Cl1	0.02137 (17)	0.02970 (19)	0.02380 (18)	−0.00376 (12)	0.00323 (12)	0.00098 (12)
S1	0.01985 (17)	0.02394 (18)	0.02535 (18)	0.00043 (12)	0.00505 (12)	0.00406 (12)
N1	0.0187 (5)	0.0228 (5)	0.0206 (5)	−0.0050 (4)	0.0009 (4)	−0.0024 (4)
N2	0.0171 (5)	0.0214 (5)	0.0217 (5)	−0.0008 (4)	0.0018 (4)	0.0005 (4)
N3	0.0193 (5)	0.0236 (5)	0.0260 (5)	0.0001 (4)	0.0024 (4)	0.0035 (4)
C1	0.0185 (5)	0.0218 (5)	0.0206 (5)	−0.0041 (4)	−0.0026 (4)	−0.0023 (4)
C2	0.0192 (5)	0.0212 (5)	0.0177 (5)	−0.0023 (4)	−0.0021 (4)	−0.0023 (4)
C11	0.0165 (5)	0.0190 (5)	0.0215 (6)	−0.0035 (4)	−0.0016 (4)	−0.0040 (4)
C12	0.0167 (5)	0.0248 (6)	0.0298 (6)	0.0024 (4)	−0.0023 (5)	0.0017 (5)
C13	0.0223 (6)	0.0259 (6)	0.0278 (6)	0.0011 (5)	−0.0031 (5)	0.0064 (5)
C14	0.0173 (5)	0.0216 (5)	0.0209 (5)	−0.0031 (4)	0.0003 (4)	−0.0032 (4)
C15	0.0148 (5)	0.0260 (6)	0.0233 (6)	−0.0005 (4)	−0.0034 (4)	−0.0027 (5)
C16	0.0183 (5)	0.0248 (6)	0.0201 (5)	−0.0016 (4)	−0.0047 (4)	−0.0010 (4)

### Geometric parameters (Å, °)

**Table:**

Cl1—C14	1.7435 (12)	C11—C16	1.3967 (16)
S1—C2	1.6992 (12)	C11—C12	1.3972 (18)
N1—C1	1.2853 (16)	C12—C13	1.3835 (18)
N1—N2	1.3824 (14)	C12—H12	0.9500
N2—C2	1.3530 (15)	C13—C14	1.3898 (17)
N2—H2	0.863 (19)	C13—H13	0.9500
N3—C2	1.3211 (16)	C14—C15	1.3851 (17)
N3—H3A	0.877 (19)	C15—C16	1.3886 (17)
N3—H3B	0.82 (2)	C15—H15	0.9500
C1—C11	1.4648 (16)	C16—H16	0.9500
C1—H1	0.9500		
			
C1—N1—N2	114.01 (10)	C13—C12—C11	120.82 (11)
C2—N2—N1	120.12 (10)	C13—C12—H12	119.6
C2—N2—H2	119.3 (12)	C11—C12—H12	119.6
N1—N2—H2	118.7 (12)	C12—C13—C14	119.15 (12)
C2—N3—H3A	118.9 (13)	C12—C13—H13	120.4
C2—N3—H3B	119.0 (13)	C14—C13—H13	120.4
H3A—N3—H3B	122.1 (18)	C15—C14—C13	121.30 (11)
N1—C1—C11	121.62 (11)	C15—C14—Cl1	119.81 (9)
N1—C1—H1	119.2	C13—C14—Cl1	118.88 (10)
C11—C1—H1	119.2	C14—C15—C16	119.00 (11)
N3—C2—N2	117.92 (11)	C14—C15—H15	120.5
N3—C2—S1	123.29 (9)	C16—C15—H15	120.5
N2—C2—S1	118.78 (9)	C15—C16—C11	120.85 (11)
C16—C11—C12	118.86 (11)	C15—C16—H16	119.6
C16—C11—C1	118.86 (11)	C11—C16—H16	119.6
C12—C11—C1	122.26 (10)		
			
C1—N1—N2—C2	−178.33 (11)	C11—C12—C13—C14	0.5 (2)
N2—N1—C1—C11	−176.64 (10)	C12—C13—C14—C15	−0.6 (2)
N1—N2—C2—N3	6.86 (18)	C12—C13—C14—Cl1	−179.11 (10)
N1—N2—C2—S1	−173.83 (8)	C13—C14—C15—C16	−0.29 (19)
N1—C1—C11—C16	−168.81 (11)	Cl1—C14—C15—C16	178.22 (9)
N1—C1—C11—C12	13.02 (19)	C14—C15—C16—C11	1.31 (18)
C16—C11—C12—C13	0.53 (19)	C12—C11—C16—C15	−1.43 (18)
C1—C11—C12—C13	178.70 (12)	C1—C11—C16—C15	−179.66 (11)

### Hydrogen-bond geometry (Å, °)

**Table:**

*D*—H···*A*	*D*—H	H···*A*	*D*···*A*	*D*—H···*A*
N2—H2···S1^i^	0.863 (19)	2.628 (19)	3.4288 (12)	154.7 (16)
N3—H3A···S1^ii^	0.877 (19)	2.65 (2)	3.5119 (12)	170.1 (17)

Symmetry codes: (i) −*x*+3, −*y*+1, −*z*; (ii) −*x*+4, −*y*, −*z*.
